# Detection and Molecular Characterization of a Canine Norovirus

**DOI:** 10.3201/eid1408.080062

**Published:** 2008-08

**Authors:** Vito Martella, Eleonora Lorusso, Niccola Decaro, Gabriella Elia, Arianna Radogna, Maria D’Abramo, Costantina Desario, Alessandra Cavalli, Marialaura Corrente, Michelle Camero, Cinzia A. Germinario, Krisztian Bányai, Barbara Di Martino, Fulvio Marsilio, Leland E. Carmichael, Canio Buonavoglia

**Affiliations:** * University of Bari, Bari, Italy; † Hungarian Academy of Sciences, Budapest, Hungary; ‡ Cornell University, Ithaca, New York, USA; § University of Teramo, Teramo, Italy

**Keywords:** dog, calicivirus, norovirus, enteritis, zoonosis, dispatch

## Abstract

We identified a novel calicivirus in a pup with enteritis. The isolate was related genetically (90.1% aa identity in the capsid protein) to a lion norovirus strain.

Caliciviridae are small, nonenveloped viruses, ≈35 nm in diameter with single-stranded, positive-polarity RNA genomes of 7.4–8.3 kb (*1*). Unlike calicivirus infections in cats, caliciviruses are not regarded as important pathogens in dogs and are not usually included in diagnostic algorithms for canine infectious diseases. Calicivirus-like particles have been occasionally identified by electron microscopy in specimens from dogs with diarrhea and, in some instances, glossitis, balanitis, or vesicular vaginitis. Most of these isolates were feline caliciviruses (*Vesivirus* genus) and were likely acquired from cats (*2*,*3*).

Thus far, only 2 documented reports have identified authentic canine caliciviruses in dogs. A calicivirus was isolated from the feces of a 4-year-old dog with bloody diarrhea and central nervous system disturbance in 1985 (Tennessee, USA). The virus replicated in experimentally infected dogs and elicited seroconversion, although disease was not reproduced. The virus was antigenically unrelated to feline calicivirus, and antibodies against the virus were identified in 76% of the canine serum specimens collected (*4*). However, the virus was not characterized molecularly, and its taxonomic status remains uncertain.

In 1990, another calicivirus was identified in Japan in a 2-month-old pup with intermittent fluid diarrhea (*5*). The virus, strain 48, was antigenically and genetically unrelated to feline calcivirus, was tentatively proposed as a “true” canine calicivirus (CaCV), and was included in the *Vesivirus* genus (*6*). Antibodies to CaCV 48 have been detected in 57% of dogs in Japan (*7*) and in 36.5% of dogs in South Korea (*8*). In this report, we describe the detection of a novel enteric CaCV.

## The Study

A gastroenteric disease with diarrhea and vomiting developed in a 60-day-old mixed-breed pup. Because of severe dehydration, the pup was hospitalized 3 days after onset of the gastroenteric symptoms. After 4 days of illness, the dog recovered completely. At the time of the dog’s hospitalization, feces were collected and screened for common canine viral pathogens; test results were positive for canine parvovirus type-2a (CPV-2a). By using a broadly reactive primer pair, p289–p290, targeted to highly conserved motives “DYSKWDST” and “YGDD” of the RdRp region of the polymerase complex, we found, unexpectedly, that stool also tested positive for calicivirus (*9*). After the detection of calicivirus RNA in the dog’s feces, the animal was kept under observation, and fecal samples were collected daily from 11 to 30 days posthospitalization (dph) to monitor virus shedding. CPV-2a DNA was detected until dph 10, whereas calicivirus RNA was detected until dph 22.

To determine the sequence and genomic organization of the novel calicivirus, a 3.4-kb region at the 3′ end of the genome was amplified by reverse transcription–PCR (RT-PCR) as described by Wang et al. (*10*), cloned into a vector, and sequenced. The sequence (3381 nt) from the 3′ end of open reading frame 1 (ORF1) to the poly-A tail of the CaCV (strain 170/07) was made available in GenBank (accession no. EU224456).

**Figure 1:**
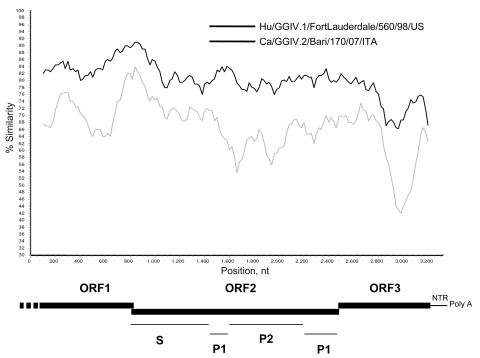
Genome organization of the canine norovirus GIV.2/Bari/170/07/ITA. The genome (from the 3′ end of open reading frame 1 [ORF1] to the poly-A tail) of the canine strain and of the human GGIV.1 norovirus, Fort Lauderdale/560/98/US (AF414426) was plotted against the genome of the strain lion/GGIV.2/Pistoia/387/06/ITA (EF450827). Sequences were analyzed with Simplot (*11*) by using a window size of 200 nt and step size of 20 nt with gap strip off and Hamming correction on. The conserved shell (S) domain and the variable regions (P1 and P2) are also indicated.

The 3.4-kb fragment of the calicivirus genome (the 3′ end of ORF1, the full-length ORF2, ORF3, and the noncoding region through the poly-A tail), is represented in Figure 1. The 3′ partial sequence of ORF1 spanned 824 nt and 273 aa at the COOH– terminus of the polymerase complex. Using BLAST (www.ncbi.nlm.nih.gov/BLAST) and FASTA (www.ebi.ac.uk/fasta33) analysis, we found the highest identity (84.6% nt and 96.7% aa) in a lion norovirus strain, Pistoia/387/06/ITA (*12*). A 14-nt overlap was present in the ORF1–ORF2 junction region, as with most described human and animal noroviruses. The ORF2 was 1737-nt long and predicted to encode for a capsid protein with a size of 578 aa. The highest sequence match was found to the lion GGIV.2 norovirus strain Pistoia/387/06/ITA (90.1% aa and 81.13% nt), while the identity was 69.4%–68.2% aa (75.5%–74.0% nt) to GIV.1 NoVs and <53.3% aa (<67.4% nt) to non–GGIV NoVs. There was a single nt overlap between ORF2 and ORF3; also, there was a 55-nt long nontranslated region between ORF3 and the poly-A tail. ORF3 was 783 nt in length and encoded for a 260-aa polypeptide.

**Figure 2:**
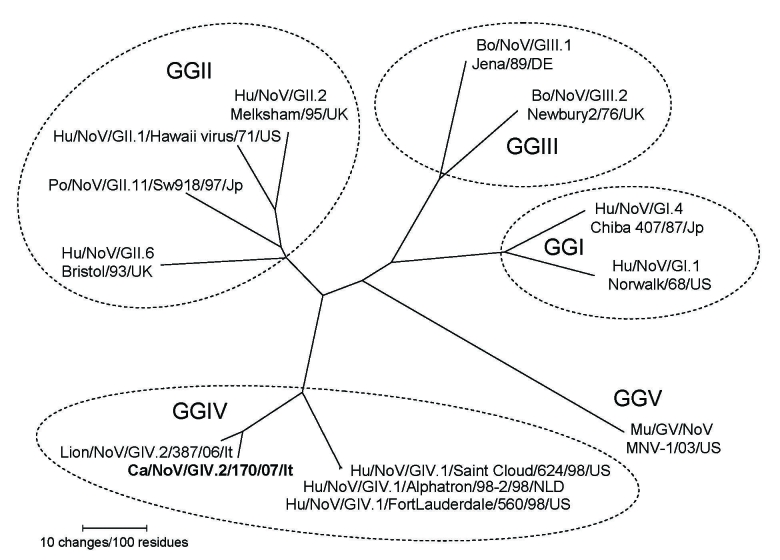
Phylogenetic tree constructed on the full-length amino acid sequence of the capsid protein. The tree was constructed by using a selection of norovirus (NoV) strains representative of the genogroups I to V. Phylogenetic analysis (neighbor-joining) with bootstrap analysis (1,000 replicates) and Kimura 2-parameter correction was conducted by using the MEGA software package version 3.0 (*14*). Strains designation follows the outlines of Wang et al. (*10*) and Zheng et al. (*13*). Bo, bovine; po, porcine; mu, murine; hu, human; ca canine.

A nucleotide identity plot of the canine norovirus genome 170/07 (from the 3′ end of ORF1 to the poly-A tail) was compared with the lion GIV.2 NoV Pistoia/387/06/ITA and the human GGIV.1 norovirus, Fort Lauderdale/560/98/US (AF414426) (Figure 1). The 3′ genome of the canine norovirus 170/07 displayed high nucleotide conservation with the lion norovirus strain. A phylogenetic tree was constructed by using the capsid protein of selected human and animal noroviruses of the various norovirus genogroups (GGI to V) (*10*,*13*). In the tree (Figure 2), the canine calicivirus (strain 170/07) was grouped with the lion GGIV.2 norovirus strain Pistoia/387/06/ITA and with the GGIV.1 human NoVs Alphatron/98–2/98/NLD, Saint Cloud/624/98/US and FortLauderdale/560/98/US.

## Conclusions

Noroviruses are regarded as the major cause of epidemic, nonbacterial gastroenteritis worldwide in humans of all age groups. The viruses are highly contagious and are transmitted by direct contact or by contaminated water and food (*15*). Because of the possibility of genetic recombination, a consistent and reliable classification of norovirus relies on the analysis of the complete capsid gene (*13*). Strains within the same genotype (or cluster) share >85% aa identity, while strains of different genotypes within the same genogroup share 55%–85% aa identity (*13*). Human noroviruses are classified into genogroups I, II, and IV. In addition, noroviruses detected in pigs, cows, and mice are classified in genogroups II, III, and V (*13*) (Table). Recently, a norovirus strain (Pistoia/387/06/ITA) was identified in a captive lion cub with severe hemorrhagic enteritis. By sequence analysis, the virus was found to resemble human GIV norovirus and was classified as a distinct genotype, GIV.2, whereas the human GIV noroviruses are genotype 1 (*12*). The canine norovirus strain 170/07 appeared to be most related genetically to the lion GGIV.2 norovirus (90.1% aa identity in the capsid protein); therefore, the virus may be considered as a variant of the genotype GGIV.2. Taken together, these findings suggest that noroviruses genetically similar to human GIV noroviruses are harbored in domestic and wild carnivores.

**Table:** Distribution of norovirus genogroups and genotypes across the various animal species

Host	Norovirus genogroups and genotypes*				
	GGI	GGII	GGIII	GGIV	GGV
Human	1–8	1–10, 12–17		1	
Pig		11, 18, 19			
Cattle			1, 2		
Lion				2†	
Dog				2‡	
Mouse					1

*Norovirus classification follows the outlines of Wang et al. (*10*) and Zheng et al. (*13*). †Martella et al. (*12*). ‡This study.

The pathogenic potential of this novel calicivirus in carnivores remains to be elucidated. In the pup, the norovirus strain was detected in conjunction with a CPV-2a strain that was likely responsible for the severity of the observed clinical signs, since CPV-2 is a major enteric pathogen of dogs. Accordingly, it is difficult to speculate on the pathogenic potential of the novel calicivirus for dogs and experimental infections in gnotobiotic dogs are required. Nevertheless, the norovirus 170/07 was detected in the subject pup for at least 22 days, which indicates active viral replication; the possibility of passive viral transit (mechanical passage) in the intestinal tract seems unlikely.

In conclusion, our study presents evidence for the existence of a novel calicivirus with enteric tropism that is related genetically to GGIV noroviruses and able to infect dogs. Future large-scale virologic and serologic investigations are needed to assess the ecology of this novel virus in wild and domestic carnivores. Also, because of the extensive social interactions between humans and pets, investigating the zoonotic potential of such animal noroviruses in humans is worthwhile.
